# Indicators of Elder Mistreatment: Correlates among Veterans Receiving Care in the Veterans Health Administration

**DOI:** 10.1093/geroni/igab046.3195

**Published:** 2021-12-17

**Authors:** Jenefer Jedele, Cameron Griffin, Julie Weitlauf

**Affiliations:** 1 Veterans Health Administration, Ann Arbor, Michigan, United States; 2 VA, Ann Arbor, Michigan, United States; 3 VAPAHCS/Stanford, Palo Alto, California, United States

## Abstract

Among community-dwelling adults ages 65 and older, approximately 11% have experienced elder mistreatment (EM), including physical, emotional or sexual abuse, neglect, or financial exploitation. EM research typically focuses on this age group; however, Veterans receiving Veterans Health Administration (VHA) care have increased earlier morbidity, which may accelerate the impacts of EM. Using a cohort of all VHA Veterans 50 years and older with VHA use in 2018-2020, we examined correlates of EM. ICD-10 codes from clinical encounters identified Veterans with indications of EM (n=4,427). A 10% sample of Veterans without indications of EM was selected for comparison (n=530,535). Logistic regression compared EM+ Veterans to the comparison sample and assessed overall demographic and clinical differences as well as differences by age, i.e. 50-64 versus 65 and older. Overall, female gender (OR=5.3, 95% CI=4.3-6.5), non-white race/ethnicity (OR=1.7, CI=1.5-1.9), dementia (OR=3.0, CI=2.6-3.5), PTSD (OR=2.0, CI=1.6-2.5), anxiety (OR=1.3, CI=1.0-1.5), military service connected disability status (OR=1.3, CI=1.1-1.5), and higher Elixhauser medical morbidity scores (OR=1.1, CI=1.1-1.1) were associated with EM. Prior year ER visits (OR=28.0, CI=23.6-33.4), inpatient stays (OR=14.0, CI=11.5-17.0), and mental health visits (OR=26.1, CI=22.2-30.6) also predicted EM+ status. Forty-six percent of VHA Veterans with indicators of EM were aged 50-64. For these Veterans, female gender, PTSD, service connection, and mental health visits were associated with increased risk of EM compared to Veterans 65+. Findings highlight clinical correlates of EMs among Veterans in VHA care. Increased awareness of EM risk factors is warranted and may inform VHA efforts for EM prevention, detection and intervention.

